# The National Childhood Cancer Registry of Chile: a population-based study of childhood cancer and insights for low- and middle-income countries

**DOI:** 10.1016/j.lana.2026.101588

**Published:** 2026-08-07

**Authors:** Patricia Cerda, Kelly Núñez, Miguel Paredes, María Paz Santana, Julia Palma, Jorge Vilches

**Affiliations:** a Department of Epidemiology, Ministry of Health, Government of Chile, Santiago, Chile; b Ministry of Health, Government of Chile, Santiago, Chile; c St. Jude Children's Research Hospital, Memphis, TN, USA

**Keywords:** Paediatric cancer, Cancer registry, Survival, Incidence, Mortality, Health policies, Chile, Latin America

## Abstract

**Background:**

Cancer registries in Latin America continue to face challenges related to coverage, under-registration, and data quality. We evaluated childhood cancer epidemiology in Chile from 2007 to 2023, assessed the quality of the National Childhood Cancer Registry (RENCI) according to International Agency for Research on Cancer (IARC) standards, and described the childhood cancer control policy context.

**Methods:**

We analysed 8821 incident cases in children aged 0–14 years registered in RENCI and classified according to ICD-O-3.2 and ICCC-3. Incidence and mortality rates were age-standardised using the Segi world standard population. Mortality and years of potential life lost (YPLL) were estimated using national mortality data (2002–2021). Trends were assessed using Joinpoint regression and five-year observed survival using the Kaplan–Meier method. Registry quality was evaluated according to IARC criteria.

**Findings:**

Incidence increased from 137.4 to 143.0 per million (AAPC 1.36, 95% CI 0.5–2.4), with leukaemias remaining the most common cancer type (58.6 per million), followed by central nervous system tumours (24.8) and lymphomas (12.7). Five-year survival improved from 71.4% (95% CI 69.6–73.8) in 2007–2011 to 80.5% (95% CI 76.4–83.9) in 2020–2023. Mortality declined from 36.3 to 25.7 per million between 2002 and 2021 (AAPC –0.57, 95% CI –1.5 to 0.4). RENCI achieved 100% national coverage, an MV% of 93.5%, and a DCO% of 0.7%.

**Interpretation:**

This study describes the implementation and operation of a national population-based childhood cancer registry in a middle-income country and reports its epidemiological findings. Key findings include increasing incidence, improved survival in recent cohorts, and a non-significant downward trend in mortality. These results offer practical insights for other low- and middle-income countries seeking to strengthen childhood cancer surveillance systems. The descriptive nature of the study and the temporal (non-causal) associations observed should be considered when interpreting the findings.

**Funding:**

National Childhood Cancer Registry (RENCI) of the Ministry of Health of Chile.

## Abstract (es)

**Antecedentes:**

Los registros de cáncer en América Latina continúan enfrentando desafíos relacionados con cobertura, subregistro y calidad de los datos. Evaluamos la epidemiología del cáncer infantil en Chile entre 2007 y 2023, analizamos la calidad del Registro Nacional de Cáncer Infantil (RENCI) de acuerdo con los estándares de la Agencia Internacional para la Investigación del Cáncer (IARC) y describimos el contexto de las políticas de control del cáncer infantil.

**Métodos:**

Analizamos 8821 casos incidentes en niños y niñas de 0 a 14 años registrados en RENCI y clasificados según ICD-O-3.2 e ICCC-3. Las tasas de incidencia y mortalidad se estandarizaron por edad utilizando la población estándar mundial de Segi. La mortalidad y los años potenciales de vida perdidos (AVPP) se estimaron utilizando datos nacionales de mortalidad correspondientes al período 2002–2021. Las tendencias se evaluaron mediante regresión Joinpoint y la supervivencia observada a cinco años mediante el método de Kaplan–Meier. La calidad del registro se evaluó de acuerdo con los criterios de la IARC.

**Resultados:**

La incidencia aumentó de 137.4 a 143.0 por millón (AAPC 1.36; IC 95%: 0.5–2.4), manteniéndose las leucemias como el tipo de cáncer más frecuente (58,6 por millón), seguidas por tumores del sistema nervioso central (24.8 por millón) y linfomas (12.7 por millón). La supervivencia a cinco años mejoró de 71.4% (IC 95%: 69.6–73.8) en el período 2007–2011 a 80.5% (IC 95%: 76.4–83.9) en 2020–2023. La mortalidad disminuyó de 36.3 a 25.7 por millón entre 2002 y 2021 (AAPC –0.57; IC 95%: –1.5 a 0.4). RENCI alcanzó una cobertura nacional del 100%, un porcentaje de verificación microscópica (MV%) de 93.5% y un porcentaje de casos identificados únicamente a partir del certificado de defunción (DCO%) de 0.7%.

**Interpretación:**

Este estudio describe la implementación y el funcionamiento de un registro nacional poblacional de cáncer infantil en un país de ingresos medios y presenta sus principales hallazgos epidemiológicos. Entre los resultados más relevantes se encuentran el aumento de la incidencia, la mejora de la supervivencia en las cohortes más recientes y una tendencia descendente no significativa de la mortalidad. Estos hallazgos ofrecen información útil para otros países de ingresos bajos y medianos que buscan fortalecer sus sistemas de vigilancia de cáncer infantil.

La naturaleza descriptiva del estudio y las asociaciones temporales observadas (no causales) deben considerarse al interpretar los hallazgos.

**Research in context**

**Evidence before this study**

We searched PubMed, Scopus, LILACS, and Google Scholar for articles published in English or Spanish between Jan 1, 2000, and Dec 31, 2024, using combinations of the terms “childhood cancer”, “paediatric cancer”, “cancer registry”, “population-based cancer registry”, “incidence”, “survival”, “mortality”, “Latin America”, and “Chile”. We also reviewed reports from the International Agency for Research on Cancer (IARC), the International Incidence of Childhood Cancer (IICC) project, the World Health Organization (WHO), and the WHO Global Initiative for Childhood Cancer (CureAll). Published evidence from Latin America remains limited and heterogeneous with respect to population coverage, data quality, and the availability of long-term survival estimates. Few studies have reported national population-based data on childhood cancer incidence, mortality, survival, and registry quality indicators within a single analytical framework.

**Added value of this study**

Using data from Chile's National Childhood Cancer Registry (RENCI), this study describes childhood cancer incidence, mortality, observed survival, and registry quality indicators at the national level between 2007 and 2023, providing a comprehensive epidemiological profile of childhood cancer in Chile. The analysis incorporates internationally recognised measures of registry quality, including microscopic verification, death certificate-only cases, mortality-to-incidence ratio, comparability, and timeliness, providing information on both childhood cancer burden and the epidemiological surveillance system that underpins these estimates. These findings contribute population-based evidence on childhood cancer epidemiology and cancer registration in Latin America.

**Implications of all the available evidence**

The findings presented here may be relevant to childhood cancer control efforts in other low- and middle-income settings, particularly in relation to population-based cancer registration and long-term epidemiological surveillance. Continued efforts to strengthen childhood cancer registration systems may improve the availability and comparability of data used to monitor disease burden and outcomes over time.

## Introduction

Paediatric cancer is a relatively rare disease; however, important inequalities persist in outcomes across countries. While survival reaches around 80% in high-income countries, it remains between 10% and 20% in low- and middle-income countries (LMICs), where approximately 80% of paediatric cancer cases occur.^1^^,^^2^ Improvements in childhood cancer outcomes have been reported in settings with strengthened access to diagnosis and treatment.^3^

Cancer registries are essential for understanding disease burden, identifying epidemiological patterns, evaluating interventions and informing public health policy. However, in Latin America, registries still face major challenges related to coverage, under-registration, and variable data quality, with population-based coverage estimated at only 16%.^4^^,^^5^

In Chile, the National Childhood Cancer Registry (RENCI) was established in 2007 to strengthen the epidemiological surveillance of paediatric cancer in Chile.^6^

It is among the few nationwide paediatric cancer registries currently operating in South America.^5^, ^6^, ^7^, ^8^ RENCI covers 100% of children aged 0–14 years (∼3.7 million), applies ICD-O-3^9^ and ICCC-3^10^ classification standards, is recognised by the International Association of Cancer Registries (IACR) and operates a platform containing 52 variables and used by 45 registered users across 24 centres nationwide (15 public and 9 private), all with secure password-protected access.

The aim of this study was to describe the epidemiology of childhood cancer in Chile, assess the quality of the National Childhood Cancer Registry (RENCI) according to International Agency for Research on Cancer (IARC) standards, and describe the policy context for childhood cancer control.

We report national trends in paediatric cancer in Chile from 2007 to 2023, assess the quality of RENCI against IARC criteria,^11^ and describe the policy context for childhood cancer control, including network-based care delivered through shared clinical protocols,^12^^,^^13^ free access to care from the point of diagnostic suspicion, and workforce training.

## Methods

### Study design and data source

This was a descriptive population-based observational study of childhood cancer in Chile. We used data from the National Childhood Cancer Registry (RENCI), a nationwide population-based registry maintained by the Department of Epidemiology of the Ministry of Health. The study population comprised all children aged 0–14 years diagnosed with cancer in Chile between 2007 and 2023. RENCI provides complete national coverage of the paediatric population (approximately 3.7 million children aged 0–14 years).

Eligible cases included incident malignant neoplasms (ICD-O-3.2 behaviour code/3), irrespective of diagnostic basis; benign and uncertain behaviour neoplasms of the central nervous system, pituitary gland, pineal gland, and craniopharyngeal duct (ICD-O-3.2 behaviour codes/0 and/1), consistent with ICCC-3 recommendations; multiple primary neoplasms registered according to IARC/IACR rules; and histiocytosis cases classified as behaviour code/3 according to the 2020 ICD-O-3.2 update. Benign and uncertain behaviour tumours outside the central nervous system and related anatomical sites were not considered eligible for inclusion.

This study was reported in accordance with the Strengthening the Reporting of Observational Studies in Epidemiology (STROBE) guidelines and the REporting of studies Conducted using Observational Routinely-collected health Data (RECORD) statement.^14^^,^^15^

### Statistical analysis

From an initial database of 9690 records, we excluded cases that did not meet the inclusion criteria: 678 duplicate records, 19 cases in children older than 15 years, 19 benign or uncertain behaviour tumours (behaviour codes/0 or/1) occurring outside the central nervous system and related sites eligible under ICCC-3, 145 cases involving foreign minors, and 8 histiocytosis cases not classified as behaviour code/3. The final analysis therefore included 8821 incident cases in children aged 0–14 years registered in RENCI between 2007 and 2023.

Incidence and survival analyses covered the period 2007–2023, corresponding to the period from the establishment of the National Childhood Cancer Registry (RENCI) to the most recent year with complete and validated data available for analysis.

Incidence was estimated annually, for the overall study period, and for the specific periods 2007–2011, 2012–2016, 2017–2019, and 2020–2023, to match the survival analysis periods.

To assess incidence by region of residence, the number of incident cases in each region was divided by the population of children aged 0–14 years during 2007–2023.

Overall incidence rates were age-standardised using the Segi world standard population (weights: 12,000 for ages 0–4 years, 10,000 for 5–9 years and 9000 for 10–14 years), a historical and widely used standard in population-based paediatric cancer registries.^16^ Denominators were based on population projections from the National Institute of Statistics (INE) of Chile.^17^

Incidence stratified by age group (0–4, 5–9 and 10–14 years), ICCC-3 diagnostic group, and sex is presented in Supplementary Material S2 as the number of incident cases and age-specific incidence rates (per million children per year).

Survival time was calculated from the date of diagnosis until death from any cause or the end of follow-up, whichever occurred first. Deaths from any cause were considered events. Cases identified solely by death certificate (n = 66) were excluded from survival analyses.

Given the national registry's systematic linkage with the national mortality database, no significant loss to follow-up was observed. Vital status was ascertained through linkage with the mortality database of the Department of Health Statistics and Information (DEIS). Patients alive at the end of the observation period were right-censored at the administrative closing date of follow-up for each diagnostic cohort.

Observed five-year survival was estimated using the Kaplan–Meier method^18^ and 95% confidence intervals for the 2007–2011 cohort (followed until 31 December 2016), the 2012–2016 cohort (followed until 31 December 2021), the 2017–2019 cohort (followed until 31 December 2024) and the 2020–2023 cohort (followed until 31 December 2024, the last year for which deaths were validated by DEIS).

Analyses were stratified by sex, age group, and ICCC-3 diagnostic category.^10^

Mortality and years of potential life lost (YPLL) were analysed for 2002–2021 using national mortality data from the Department of Health Statistics and Information (DEIS), reflecting the longest period consistently available at the national level and allowing the assessment of long-term mortality trends over two decades, with population denominators obtained from the National Institute of Statistics (INE).^17^ Age-specific crude mortality rates (CMR, per 1,000,000) and age-standardised mortality rates (ASMR, per 1,000,000) for childhood cancer in children aged 0–14 years were calculated for 2002–2021. Crude rates were obtained by dividing the number of deaths by the population in each age group (0–4, 5–9, 10–14 years) and sex and multiplying by 1,000,000. Age-standardised rates were calculated using the direct method and the Segi world standard population (1960),^16^ with weights of 0.387 for ages 0–4 years, 0.323 for 5–9 years, and 0.290 for 10–14 years, consistent with the standard used for incidence rates. The use of the Segi standard for mortality,^16^ consistent with the standard applied to incidence, improves internal and international comparability. Ninety-five percent confidence intervals (95% CI) for both crude and age-standardised rates were calculated using the Poisson approximation for rare events.^19^

In paediatric cancer, years of potential life lost (YPLL) provide a complementary measure to mortality by quantifying the impact of deaths occurring at very young ages. However, in populations restricted to children aged 0–14 years, YPLL remain highly correlated with mortality and should therefore be interpreted with caution as a complementary rather than an independent measure of disease burden.^20^ YPLL were defined as the difference between a reference life expectancy and the age at death for each child. Following standard methodology for paediatric populations,^21^^,^^22^ reference life expectancies were set at 74.5 years for children aged 0–4 years, 69.5 years for those aged 5–9 years, and 64.5 years for those aged 10–14 years.

Temporal trends in incidence, mortality, and years of potential life lost (YPLL) were analysed using Joinpoint Regression. Log-linear models were fitted to annual rates, allowing a maximum of three joinpoints. The optimal model was selected using permutation tests. Trends are presented as annual percent change (APC) and average annual percent change (AAPC), with 95% confidence intervals (CI). Statistical significance was defined as p < 0.05.^23^

The process of case identification, initial validation, linkage with complementary data sources (DEIS death registry, hospital discharges records, SIGGES, civil registry queries and historical RENCI queries), data enrichment, resolution of inconsistencies, coding according to ICD-O-3.2 and ICCC-3, application of inclusion criteria, consolidation of the final database and quality control using data review procedures and IARC tools is illustrated in Supplementary Material S1.

### RENCI quality indicators

RENCI quality was assessed according to criteria established by the International Agency for Research on Cancer (IARC).^11^^,^^24^, ^25^, ^26^, ^27^ Validity was evaluated using the diagnostic basis indicator, including the percentage of microscopically verified cases (MV%; based on cytology, histology of the primary tumour or metastasis), the percentage of death certificate-only cases (DCO%) and the percentage of cases recorded as unspecified tumour.^25^

Completeness was assessed through population coverage (100%),^26^^,^^27^ the contribution of cases from each reporting facility since the registry was established, and cross-linkage of the RENCI database with multiple information sources, including hospital discharge databases, mortality records, population-based and hospital cancer registries, and the Explicit Health Guarantees Management Information System (SIGGES), to identify potentially unreported cases.

The mortality-to-incidence (M:I) ratio was calculated by dividing the number of deaths by the number of incident cases for the period 2007–2021, corresponding to the last year validated by DEIS. An M:I ratio below 1 indicates that mortality is lower than incidence. Given the high quality and completeness of mortality registration in Chile,^28^ the M:I ratio was considered suitable for descriptive analyses.^11^^,^^25^

To assess comparability, the registry used the ICCC-3^10^^,^^29^^,^^30^ and ICD-O^9^ classification and coding systems, defined registrable cases according to (0–14 years) and year of incidence, and evaluated the temporal stability and consistency of the distribution of paediatric cancer types between 2007 and 2021. Comparability was assessed through the use of standardised coding systems, consistent case definitions, and evaluation of temporal stability in the distribution of paediatric cancer types.^4^

Timeliness, defined as the interval between year of diagnosis and publication, was assessed as the number of years between the last incorporated case (closure year) and the year of publication of each report.^25^^,^^27^ IARC-Tools were used to verify codes and identify duplicate records.^31^

### Policy context and childhood cancer control in Chile

The policy context for childhood cancer control in Chile was described narratively, including the implementation of network-based care, standardised clinical protocols, universal access to diagnosis and treatment, and workforce training initiatives.

Descriptive statistical analyses were conducted using IBM SPSS Statistics version 26.0 (IBM Corp., Armonk, NY, USA). Observed survival analyses were performed using Stata version 18.0 (StataCorp LLC, College Station, TX, USA). Joinpoint analyses were conducted using the Joinpoint Regression Program version 5.4.0 (National Cancer Institute, Bethesda, MD, USA). Data management, graphical outputs, and descriptive tabulations were performed using Microsoft Excel (Microsoft Corporation, Redmond, WA, USA).

### Ethical considerations

This study is based on secondary analysis of anonymised and aggregated data from the National Childhood Cancer Registry (RENCI), maintained by the Department of Epidemiology of the Ministry of Health of Chile as part of mandatory public health epidemiological surveillance activities. Under current Chilean regulations, including Law No. 20.584,^32^ which regulates the rights and duties of individuals in relation to healthcare-related actions, and Law No. 20.120,^33^ which governs scientific research involving human beings, analyses of routinely collected data for epidemiological surveillance do not require individual ethical approval or additional informed consent, provided that the data are anonymised and the analysis is conducted to support improvements in the health system. Access to and use of these data are regulated by the Ministry of Health's internal rules on protection of sensitive data and confidentiality, in accordance with Law No. 19.628, as amended.^34^ The protocol for this analysis was reviewed and approved by the management team of the Department of Epidemiology of the Ministry of Health of Chile within the framework of RENCI surveillance functions. No additional risks to individuals or conflicts of interest were identified.

### Role of the funding source

This study received no specific funding. No funding body had any role in the study design, data collection, data analysis, data interpretation, writing of the report, or the decision to submit the manuscript for publication.

## Results

### Epidemiology of paediatric cancer

Of the 8821 cases included in the analysis, 4833 (54.8%) were boys, 3731 (42.3%) were children aged 0–4 years and 7201 (81.6%) were treated in the public health system. The regions of Chile with incidence rates above the national average were Los Ríos, Biobío, Antofagasta, Atacama, and La Araucanía (Table 1).

**Table 1:** Sociodemographic characteristics, tumour type, behaviour, and regional distribution of childhood cancer cases in children aged 0–14 years. Chile, 2007–2023 (RENCI).

Characteristic	Incident cases	%
Sex		
Boys	4833	54.8
Girls	3988	45.2
Age group		
0–4	3731	42.3
5–9	2398	27.2
10–14	2692	30.5
Health system		
Public	7201	81.6
Private	1620	17.4
Health coverage		
FONASA (Fondo Nacional de Salud)	7178	81.4
ISAPRE (Institución de Salud Previsional, private)	1250	14.2
DIPRECA (Dirección de Previsión de Carabineros de Chile)	63	0.7
PRAIS (Programa de Reparación y Atención Integral en Salud y Derechos Humanos)	24	0.3
CAPREDENA (Caja de Previsión de la Defensa Nacional, Fuerzas Armadas) and SISA (Seguro de Invalidez y Sobrevivencia)	33	0.4
None	54	0.6
Unknown	194	2.2
Type of neoplasia		
Primary	8803	99.8
Primary Multiple	18	0.2
Neoplasia Behaviour		
Benign	53	0.6
Uncertain	325	3.7
Malignant	8443	95.7
Regional distribution (North to South)		Crude incidence rate∗
Arica y Parinacota	116	132.7
Tarapacá	168	123.3
Antofagasta	354	148.4
Atacama	170	141.9
Coquimbo	383	135.2
Valparaíso	830	134.5
Metropolitana de Santiago	3448	136.7
Libertador Bernardo OHiggins	446	132.6
Maule	491	129.4
Biobío	1156	157.8
La Araucanía	504	140.9
Los Ríos	220	160.0
Los Lagos	417	134.3
Aysén	45	108.6
Magallanes y Antártica Chilena	72	126.7
Unknown	1	–
Total	8821	138.7

∗ Rate per 1 million children under 15 years of age.

**Fig. 1:**
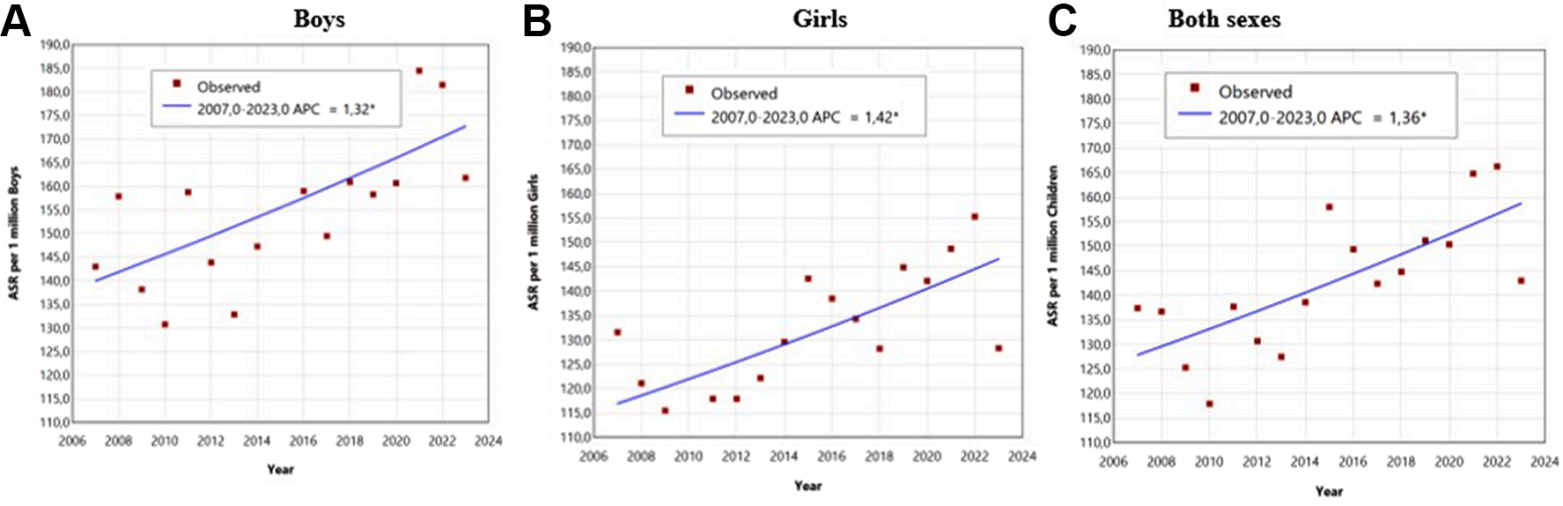
General trends in childhood cancer incidence in Chile by sex: A) Boys, B) Girls, and C) Both sexes. Note: The x-axis represents the year of incidence (2007–2023), and the y-axis shows the age-standardised incidence rate (ASR) per million children under 15 years (Segi world standard). Blue lines indicate the annual percent change (APC) from Joinpoint regression analysis. Asterisks (∗) denote APC values that are statistically significantly different from zero (p < 0.05), indicating a non-random increase or decrease in incidence rates.

The overall age-standardised incidence rate was 142.4 per million (95% CI 139.5–145.3), rising from 137.4 per million in 2007 to 143.0 per million in 2023 (AAPC 1.36, 95% CI 0.5–2.4; p < 0.05), with higher rates observed in boys (155.6 per million) than in girls (130.9 per million). Analysis by reporting periods also showed a progressive increase, with rates of 126.5, 137.9, 142.3, and 152.8 per million children aged 0–14 years in 2007–2011, 2012–2016, 2017–2019, and 2020–2023, respectively (Table 2, Fig. 1).

**Table 2:** Distribution of new cases of childhood cancer and incidence rates by sex and year.

Year	Boys				Girls				Both sexes				
	New cases	Population	Crude rate	ASR (95% CI)	New cases	Population	Crude rate	ASR (95% CI)	New cases	Population	Crude rate	ASR (95% CI)	Crude rate by period
2007	268	1,967,441	136.2	143.0 (126.6–159.3)	239	1,896,887	126.0	131.6 (115.6–147.6)	507	3,864,328	131.2	137.4 (126.0–148.8)	126.5 (121.4–131.6)
2008	291	1,946,510	149.5	157.9 (140.7–175.1)	218	1,876,573	116.2	121.1 (105.6–136.5)	509	3,823,083	133.1	136.7 (125.1–148.2)	
2009	250	1,930,887	129.5	138.2 (122.1–154.2)	207	1,861,372	111.2	115.5 (100.4–130.7)	457	3,792,259	120.5	125.3 (114.3–136.4)	
2010	237	1,918,478	123.5	130.8 (115.1–146.6)	190	1,849,289	102.7	107.0 (92.4–121.6)	427	3,767,767	113.3	117.9 (107.2–128.7)	
2011	293	1,908,919	153.5	158.8 (141.2–176.4)	210	1,840,014	114.1	117.9 (102.5–133.4)	503	3,748,933	134.2	137.7 (126.0–149.4)	
2012	264	1,900,364	138.9	143.9 (127.1–160.6)	214	1,831,741	116.8	117.9 (102.2–133.6)	478	3,732,105	128.1	130.7 (119.2–142.2)	137.9 (132.6–143.3)
2013	249	1,890,242	131.7	132.9 (116.6–149.3)	219	1,822,184	120.2	122.2 (106.3–138.1)	468	3,712,426	126.1	127.5 (116.1–138.9)	
2014	272	1,883,249	144.4	147.3 (130.1–164.4)	232	1,815,680	127.8	129.6 (113.1–146.0)	504	3,698,929	136.3	138.6 (126.7–150.5)	
2015	317	1,881,507	168.5	172.3 (153.8–190.9)	252	1,814,249	138.9	142.6 (125.4–159.7)	569	3,695,756	154.0	158.0 (145.4–170.7)	
2016	293	1,879,883	155.9	159.0 (141.2–176.9)	244	1,812,868	134.6	138.5 (121.7–155.4)	537	3,692,751	145.4	149.4 (137.1–161.7)	
2017	271	1,878,399	144.3	149.5 (132.3–166.6)	237	1,811,303	130.8	134.3 (117.7–151.0)	508	3,689,702	137.7	142.4 (130.4–154.3)	142.3 (135.2–149.3)
2018	295	1,881,765	156.8	160.9 (143.0–178.8)	227	1,814,375	125.1	128.2 (111.9–144.5)	522	3,696,140	141.2	144.8 (132.7–157.0)	
2019	289	1,891,178	152.8	158.3 (140.7–175.9)	260	1,822,994	142.6	144.9 (127.5–162.2)	549	3,714,172	147.8	151.2 (138.8–163.6)	
2020	292	1,903,242	153.4	160.7 (143.1–178.3)	255	1,834,796	139.0	142.1 (125.1–159.2)	547	3,738,038	146.3	150.4 (138.1–162.6)	152.8 (146.5–159.0)
2021	331	1,907,200	173.6	184.5 (165.8–203.2)	271	1,838,465	147.4	148.7 (131.1–166.2)	602	3,745,665	160.7	164.8 (152.0–177.6)	
2022	327	1,904,127	171.7	181.5 (162.8–200.1)	281	1,835,239	153.1	155.3 (137.4–173.2)	608	3,739,366	162.6	166.3 (153.4–179.2)	
2023	294	1,895,867	155.1	161.8 (144.1–179.5)	232	1,827,000	127.0	128.3 (112.0–144.7)	526	3,722,867	141.3	143.0 (130.9–155.1)	
**Total**	**4833**	**32,369,258**	**149.3**	**155.6 (151.4**–**159.8)**	**3988**	**31,205,029**	**127.8**	**130.9 (126.9**–**134.8)**	**8821**	**63,574,287**	**138.8**	**142.4 (139.5**–**145.3)**	138.8 (135.9–141.6)

National Childhood Cancer Registry (RENCI) Chile, 2007–2023. Crude rate and ASR per 1,000,000 children under 15 years of age.

ASR = age-standardised rate (Segi world standard population).

Leukaemias were the most common cancer type (ASR 58.6 per million; 40.4% of cases), followed by central nervous system tumours (24.8 per million, 17.6%) and lymphomas (12.7 per million, 9.5%), with higher incidence in boys, showing notable differences in leukaemias (ASR 63.9 vs. 53.0) and lymphomas (16.9 vs. 8.3) (Table 3).

**Table 3:** Incidence of childhood cancer according to ICCC-3 and sex.

	Cancer group (ICCC-3)	Boys				Girls				Both sexes			
		Cases	Crude rate∗	ASR (95% CI)	%	Cases	Crude rate∗	ASR (95% CI)	%	Cases	Crude rate∗	ASR (95% CI)	%
I	Leukaemias, myeloproliferative diseases, and myelodysplastic diseases	1979	61.1	63.9 (61.2–66.6)	40.9	1583	50.7	53.0 (50.6–55.4)	39.7	3562	56.0	58.6 (56.7–60.4)	40.4
II	Lymphomas and reticuloendothelial neoplasms	566	17.5	16.9 (15.4–18.3)	11.7	270	8.7	8.3 (7.3–9.3)	6.8	836	13.1	12.7 (11.8–13.6)	9.5
III	CNS and miscellaneous intracranial and intraspinal neoplasms	825	25.5	26.0 (24.2–27.7)	17.1	725	23.2	23.6 (22.0–25.3)	18.2	1550	24.4	24.8 (23.6–26.0)	17.6
IV	Neuroblastoma and other peripheral nervous cell tumours	174	5.4	6.2 (5.4–7.0)	3.6	143	4.6	5.2 (4.5–6.0)	3.6	317	5.0	5.7 (5.2–6.3)	3.6
V	Retinoblastoma	124	3.8	4.5 (3.8–5.2)	2.6	123	3.9	4.7 (4.0–5.3)	3.1	247	3.9	4.6 (4.1–5.1)	2.8
VI	Renal tumours	138	4.3	4.8 (4.1–5.6)	2.9	182	5.8	6.6 (5.8–7.4)	4.6	320	5.0	5.7 (5.2–6.3)	3.6
VII	Hepatic tumours	108	3.3	3.7 (3.1–4.4)	2.2	69	2.2	2.5 (2.0–3.0)	1.7	177	2.8	3.1 (2.7–3.5)	2.0
VIII	Malignant bone tumours	248	7.7	7.0 (6.0–8.0)	5.1	238	7.6	7.0 (6.0–7.9)	6.0	486	7.6	7.0 (6.3–7.7)	5.5
IX	Soft tissue and other extraosseous sarcomas	292	9.0	9.2 (8.2–10.3)	6.0	226	7.2	7.3 (6.4–8.3)	5.7	518	8.1	8.3 (7.6–9.0)	5.9
X	Germ cell tumours, trophoblastic tumours, and neoplasms of gonads	256	7.9	7.8 (6.9–8.8)	5.3	167	5.4	5.1 (4.3–5.9)	4.2	423	6.7	6.5 (5.9–7.1)	4.8
XI	Other malignant epithelial neoplasms and malignant melanomas	104	3.2	2.8 (2.2–3.5)	2.2	241	7.7	6.8 (5.9–7.7)	6.0	345	5.4	4.8 (4.2–5.4)	3.9
XII	Other and unspecified malignant neoplasms	19	0.6	0.6 (0.4–0.9)	0.4	21	0.7	0.6 (0.4–0.9)	0.5	40	0.6	0.6 (0.4–0.8)	0.5
	Total	4833	**149.3**	**155.6 (151.4**–**159.8)**	100.0	3988	**127.8**	**130.9 (127.0**–**134.7)**	100.0	8821	**138.8**	**142.4 (139.5**–**145.3)**	100.0

Chile, 2007–2023.

ASR = age-standardised rate (Segi world standard population).

∗ Crude rate and ASR per 1,000,000 children under 15 years of age.

Leukaemias predominated as the most common neoplasm across all age groups and both sexes, with the highest age-specific incidence rate observed in boys aged 0–4 years (88.7 per million), followed by girls in the same age group (73.2 per million). Age-specific incidence rates for leukaemias declined with age, from 81.1 per million in children aged 0–4 years to 39.1 per million in those aged 10–14 years, whereas rates increased with age for lymphomas (from 8.6 to 16.4 per million) and malignant bone tumours (from 2.3 to 14.1 per million). The detailed distribution of incident cases and incidence rates by ICCC-3 diagnostic group, sex and age subgroup is presented in Supplementary Material S2.

Five-year survival improved from 71.4% (95% CI 69.6–73.8) in 2007–2011 to 80.5% (95% CI 76.4–83.9) in 2020–2023, with higher rates in girls (83.7% vs. 77.8% in boys) and in the 10–14 years age group (82.4%, 95% CI 79.2–85.2).

Notable improvements in five-year survival were observed for retinoblastoma increasing from 82.4% (95% CI 71.5–89.5) in 2007–2011 to 100% in 2017–2019 and 2020–2023, and for hepatic tumours, increasing from 59.0% [95% CI 42.8–72.0] in 2007–2011 to 86.5% [95% CI 72.5–93.7] in 2020–2023, as detailed in Table 4.

**Table 4:** Trends in five-year survival from childhood cancer by sex, age group, and ICCC-3 diagnostic group (Kaplan–Meier estimates with 95% confidence intervals), Chile: National Childhood Cancer Registry (RENCI), 2007–2011 to 2020–2023.

Analysis group	Year of incidence			
	2007–2011	2012–2016	2017–2019	2020–2023
	% (95% CI)	% (95% CI)	% (95% CI)	% (95% CI)
Sex				
Both sexes	**71.4** (69.3–73.5)	**73.5** (71.3–75.5)	**78.5** (76.2–80.8)	**80.5** (76.4–83.9)
Boys	**69.6** (66.6–72.4)	**74.5** (71.6–77.0)	**77.9** (74.5–80.8)	**77.8** (70.6–83.4)
Girls	**73.6** (70.4–76.6)	**72.3** (68.9–75.4)	**78.9** (75.7–81.8)	**83.7** (80.8–86.1)
Age group (years)				
0–4	**72.5** (69.2–75.5)	**75.1** (71.2–78.0)	**78.1** (74.5–81.3)	**77.2** (66.6–84.8)
5–9	**77.6** (74.0–80.8)	**72.4** (68.1–76.2)	**79.0** (74.7–82.7)	**82.2** (77.3–86.2)
10–14	**74.2** (70.6–77.5)	**72.3** (68.2–76.1)	**78.1** (73.6–81.9)	**82.4** (79.2–85.2)
ICCC-3 diagnostic group				
I. Leukaemias, myeloproliferative diseases, and myelodysplastic diseases	**71.6** (68.0–74.9)	**74.4** (70.9–77.6)	**78.3** (74.7–81.5)	**79.9** (74.7–84.1)
II. Lymphomas and reticuloendothelial neoplasms	**83.9** (76.5–89.1)	**85.8** (80.0–90.0)	**91.4** (85.6–94.9)	**95.3** (91.1–97.6)
III. CNS and miscellaneous intracranial and intraspinal neoplasms	**58.7** (52.8–64.0)	**59.7** (54.1–64.9)	**67.8** (61.6–73.2)	**77.1** (71.4–81.7)
IV. Neuroblastoma and other peripheral nervous cell tumours	**68.8** (57.7–77.6)	**62.1** (46.4–74.4)	**82.8** (70.3–90.3)	**74.4** (59.6–84.4)
V. Retinoblastoma	**82.4** (71.5–89.5)	**94.2** (85.2–97.8)	**100.0** (100.0–100.0)	**100.0** (100.0–100.0)
VI. Renal tumours	**83.8** (74.1–90.1)	**88.8** (80.0–93.9)	**85.2** (71.3–92.7)	**60.3** (8.3–90.2)∗
VII. Hepatic tumours	**59.0** (42.8–72.0)	**58.5** (43.9–70.5)	**70.5** (34.0–89.3)	86.5 (72.5–93.7)
VIII. Malignant bone tumours	**65.3** (54.1–74.4)	**64.7** (54.4–73.3)	**60.6** (48.3–70.8)	**65.7** (53.4–75.4)
IX. Soft tissue and other extraosseous sarcomas	**63.8** (55.0–71.3)	**63.3** (53.5–71.5)	**73.1** (60.8–82.1)	**74.1** (65.6–80.7)
X. Germ cell tumours, trophoblastic tumours, and neoplasms of gonads	**82.7** (72.5–89.3)	**88.3** (80.9–93.0)	**88.2** (78.5–93.7)	**91.3** (82.0–95.9)
XI. Other malignant epithelial neoplasms and malignant melanomas	**81.6** (67.9–89.8)	**98.7** (91.1–99.8)	**94.4** (85.7–97.9)	**98.5** (94.0–99.6)
XII. Other and unspecified malignant neoplasms	–	–	–	**80.1** (54.2–92.2)
Total	**71.4** (69.3–73.5)	**73.5** (71.3–75.5)	**78.5** (76.2–80.8)	**80.5** (76.4–83.9)

Kaplan–Meier estimates.

Five-year survival for the 2020–2023 cohort should be interpreted with caution, given the variable and incomplete follow-up in this group (ranging from 1 to approximately 5 years).

∗ The wide confidence interval observed for renal tumours in the 2020–2023 cohort is likely related to incomplete follow-up and the limited number of patients with sufficient observation time at study closure. The estimate should be interpreted with caution.

In the most recent period analysed, the highest survival estimates were observed for retinoblastoma (100%), epithelial neoplasms and malignant melanoma (98.5%, 95% CI 94.0–99.6), and lymphomas (95.3%, 95% CI 91.1–97.6). The lowest survival estimates were found for renal tumours (60.3%, 95% CI 8.3–90.2) and bone tumours (65.7%, 95% CI 53.4–75.4) (Table 4).

**Fig. 2:**
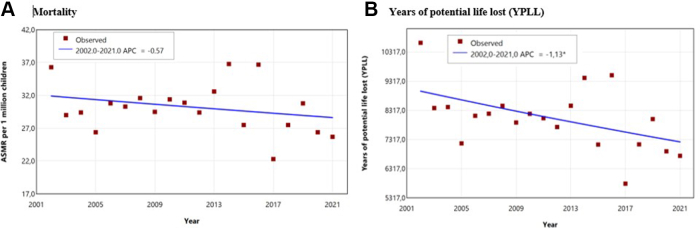
Trends in childhood cancer mortality and years of potential life lost (YPLL) in Chile (2002–2021), for both sexes combined. A) Age-standardised mortality rates (ASMR) (per million children under 15 years). B) Years of potential life lost (YPLL) attributable to childhood cancer deaths. Note: The x-axis represents the year of death (2002–2021). The y-axis shows the standardised mortality rate (per million children aged 0–14 years) in panel A, and the total cumulative YPLL in panel B. The blue line indicates the fitted trend using Joinpoint regression analysis, representing the estimated annual percent change (APC). Asterisks (∗) indicate that the annual percent change (APC) is statistically significant (p < 0.05), meaning that the observed trend (increase or decrease) is unlikely to be due solely to random variation and suggests a real change in mortality or in the amount of premature mortality captured by YPLL during the study period. Data are derived from the National Childhood Cancer Registry (RENCI) with linkage to death certificates (DEIS).

Mortality showed a non-significant downward trend (AAPC −0.57, 95% CI −1.5 to 0.4), with an endpoint-to-endpoint change from 36.3 to 25.7 per million between 2002 and 2021. Mortality rates and years of potential life lost (YPLL) were consistently higher in boys (Table 5, Fig. 2).

**Table 5:** Evolution of mortality and premature mortality in children under 15 years of age diagnosed with cancer by year of death.

Year	Boys						Girls						Both sexes					
	Deaths boys	Population boys	CMR (95% CI)	ASMR∗ (95% CI)	YPLL	YPLL rate	Deaths girls	Population girls	CMR (95% CI)	ASMR∗ (95% CI)	YPLL	YPLL rate	Deaths total	Population total	CMR (95% CI)	ASMR∗ (95% CI)	YPLL	YPLL rate
2002	88	2,115,812	41.6 (33.8–50.4)	41.1 (39.9–42.2)	6061.0	2864.6	67	2,041,000	32.8 (25.3–41.7)	31.4 (30.3–32.4)	4566.5	2237.4	155	4,156,812	37.3 (31.8–43.5)	36.3 (35.5–37.1)	10,627.5	2556.6
2003	67	2,095,278	32.0 (25.3–40.3)	30.5 (29.4–31.5)	4576.5	2184.2	55	2,020,910	27.2 (20.6–35.4)	27.4 (26.4–28.4)	3817.5	1889.0	122	4,116,188	29.6 (24.7–35.3)	29.0 (28.3–29.7)	8394.0	2039.3
2004	68	2,065,584	32.9 (26.0–41.3)	31.4 (30.3–32.4)	4641.0	2246.8	55	1,992,045	27.6 (21.0–35.8)	27.3 (26.3–28.3)	3787.5	1901.3	123	4,057,629	30.3 (25.3–36.0)	29.4 (28.6–30.1)	8428.5	2077.2
2005	65	2,032,009	32.0 (25.1–40.3)	33.1 (32.0–34.1)	4547.5	2237.9	38	1,959,467	19.4 (14.0–26.5)	19.5 (18.7–20.3)	2636.0	1345.3	103	3,991,476	25.9 (21.3–31.2)	26.4 (25.7–27.1)	7183.5	1799.7
2006	69	1,996,477	34.6 (27.3–43.5)	36.1 (35.0–37.2)	4840.5	2424.5	47	1,925,044	24.4 (18.3–32.2)	25.3 (24.3–26.2)	3286.5	1707.2	116	3,921,521	29.7 (24.7–35.4)	30.8 (30.1–31.5)	8127.0	2072.4
2007	71	1,967,441	36.1 (28.6–45.2)	34.9 (33.7–36.0)	4864.5	2472.5	48	1,896,887	25.3 (19.0–33.2)	25.5 (24.5–26.5)	3336.0	1758.7	119	3,864,328	31.0 (25.9–36.8)	30.3 (29.5–31.0)	8200.5	2122.1
2008	73	1,946,510	37.5 (29.9–46.7)	37.2 (36.0–38.4)	5043.5	2591.0	50	1,876,573	26.6 (20.1–34.8)	25.7 (24.7–26.7)	3425.0	1825.1	123	3,823,083	32.2 (27.0–38.1)	31.6 (30.8–32.3)	8468.5	2215.1
2009	68	1,930,887	35.2 (27.8–44.2)	33.6 (32.5–34.7)	4636.0	2401.0	47	1,861,372	25.3 (19.0–33.2)	25.2 (24.3–26.2)	3261.5	1752.2	115	3,792,259	30.3 (25.3–36.0)	29.5 (28.7–30.2)	7897.5	2082.5
2010	65	1,918,478	33.9 (26.6–42.6)	34.2 (33.1–35.3)	4527.5	2359.9	53	1,849,289	28.7 (21.9–37.0)	28.5 (27.4–29.5)	3668.5	1983.7	118	3,767,767	31.3 (26.2–37.1)	31.4 (30.7–32.2)	8196.0	2175.3
2011	62	1,908,919	32.5 (25.4–41.0)	32.0 (30.9–33.1)	4274.0	2239.0	54	1,840,014	29.3 (22.4–37.8)	29.8 (28.7–30.8)	3773.0	2050.5	116	3,748,933	30.9 (25.9–36.7)	30.9 (30.1–31.7)	8047.0	2146.5
2012	66	1,900,364	34.7 (27.3–43.7)	33.8 (32.7–35.0)	4527.0	2382.2	47	1,831,741	25.7 (19.3–33.7)	24.8 (23.9–25.8)	3216.5	1756.0	113	3,732,105	30.3 (25.3–36.0)	29.4 (28.7–30.2)	7743.5	2074.8
2013	66	1,890,242	34.9 (27.5–43.9)	34.6 (33.5–35.8)	4567.0	2416.1	57	1,822,184	31.3 (24.1–40.2)	30.4 (29.3–31.5)	3906.5	2143.9	123	3,712,426	33.1 (27.8–39.2)	32.6 (31.8–33.3)	8473.5	2282.5
2014	82	1,883,249	43.5 (35.1–53.5)	43.5 (42.2–44.7)	5699.0	3026.2	53	1,815,680	29.2 (22.3–37.7)	29.9 (28.8–30.9)	3728.5	2053.5	135	3,698,929	36.5 (30.9–43.0)	36.8 (36.0–37.6)	9427.5	2548.7
2015	60	1,881,507	31.9 (24.9–40.2)	31.0 (29.9–32.1)	4110.0	2184.4	44	1,814,249	24.3 (18.1–31.9)	23.8 (22.8–24.8)	3033.0	1671.8	104	3,695,756	28.1 (23.3–33.7)	27.5 (26.7–28.2)	7143.0	1932.8
2016	76	1,879,883	40.4 (32.4–50.0)	39.7 (38.4–40.9)	5232.0	2783.2	62	1,812,868	34.2 (26.7–43.4)	33.6 (32.4–34.7)	4284.0	2363.1	138	3,692,751	37.4 (31.7–43.9)	36.7 (35.8–37.5)	9516.0	2576.9
2017	40	1,878,399	21.3 (15.6–28.9)	20.5 (19.6–21.4)	2735.0	1456.0	44	1,811,303	24.3 (18.1–31.9)	24.3 (23.3–25.3)	3068.0	1693.8	84	3,689,702	22.8 (18.4–27.9)	22.3 (21.7–23.0)	5803.0	1572.8
2018	50	1,881,765	26.6 (20.2–34.9)	26.3 (25.3–27.3)	3460.0	1838.7	54	1,814,375	29.8 (22.8–38.3)	28.8 (27.7–29.8)	3688.0	2032.7	104	3,696,140	28.1 (23.3–33.7)	27.5 (26.8–28.3)	7148.0	1933.9
2019	56	1,891,178	29.6 (22.9–37.9)	29.2 (28.2–30.3)	3882.0	2052.7	60	1,822,994	32.9 (25.5–41.8)	32.3 (31.2–33.4)	4135.0	2268.2	116	3,714,172	31.2 (26.2–37.0)	30.8 (30.0–31.5)	8017.0	2158.5
2020	55	1,903,242	28.9 (22.2–37.2)	29.0 (28.0–30.1)	3827.5	2011.0	45	1,834,796	24.5 (18.3–32.2)	23.6 (22.6–24.6)	3082.5	1680.0	100	3,738,038	26.8 (22.1–32.2)	26.4 (25.7–27.1)	6910.0	1848.6
2021	48	1,907,200	25.2 (19.0–33.1)	25.1 (24.2–26.1)	3326.0	1743.9	50	1,838,465	27.2 (20.6–35.4)	26.3 (25.3–27.3)	3430.0	1865.7	98	3,745,665	26.2 (21.5–31.5)	25.7 (25.0–26.4)	6756.0	1803.7
**Total**	**1295**	**38,874,424**	**33.3 (31.5**–**35.1)**	**32.8 (32.6**–**33.1)**	**89,377.5**	**2299.1**	**1030**	**37,481,256**	**27.5 (25.8**–**29.1)**	**27.1 (26.9**–**27.4)**	**71,130.0**	**1897.7**	**2325**	**76,355,680**	**30.5 (30.4**–**31.7)**	**30.0 (29.9**–**30.2)**	**160,507.5**	**2102.1**

Mortality rate and YPLL rate. Chile, 2002–2021.

CMR: Crude Mortality Rate.

ASMR: Age-Standardised Mortality Rate.

YPLL: Years of Potential Life Lost (rate per million).

∗ Using the Segi world standard population.

### Quality criteria of RENCI

During 2007–2023, the National Childhood Cancer Registry (RENCI) of Chile met the quality standards established by the International Agency for Research on Cancer (IARC). Completeness was supported by 100% population coverage, whereas validity was supported by a microscopically verified rate (MV%) of 93.5%, a death certificate-only rate (DCO%) of 0.7%, and only 0.5% of cases classified as unspecified tumours (Table 6).

**Table 6:** Distribution of cases according to diagnostic group (ICCC-3) and basis of diagnosis.

Diagnostic group (ICCC-3)	Basis of diagnosis codes								
					Microscopic verification (MV)				
	0	1	2	4	5	6	7	9	Total
	Death certificate only (DCO)	Clinical	Clinical investigation	Specific tumour markers (biochemical and/or immunologic markers)	Cytology	Histology of metastasis	Histology of primary site	Unknown	
I. Leukaemias, myeloproliferative diseases, and myelodysplastic diseases	24	0	0	4	3308	3	223	0	3562
II. Lymphomas and reticuloendothelial neoplasms	3	0	5	1	87	0	740	0	836
III. CNS and miscellaneous intracranial and intraspinal neoplasms	22	4	339	12	19	1	1153	0	1550
IV. Neuroblastoma and other peripheral nervous cell tumours	0	1	12	2	7	5	290	0	317
V. Retinoblastoma	0	1	54	4	5	0	183	0	247
VI. Renal tumours	1	0	16	0	4	0	299	0	320
VII. Hepatic tumours	0	0	2	1	2	0	172	0	177
VIII. Malignant bone tumours	4	1	1	1	3	1	475	0	486
IX. Soft tissue and other extraosseous sarcomas	1	0	9	1	5	3	499	0	518
X. Germ cell tumours, trophoblastic tumours, and neoplasms of gonads	1	0	7	12	4	1	398	0	423
XI. Other malignant epithelial neoplasms and malignant melanomas	2	2	4	0	5	1	331	0	345
XII. Other and unspecified malignant neoplasms	8	2	8	0	1	0	20	1	40
Total	66	11	457	38	3450	15	4783	1	8821
Percentage (%)	0.7%	0.1%	5.2%	0.4%	**39.1%**	**0.2%**	**54.2%**	0.0%	100.0%
Microscopically verified percentage (MV%)					***93.5%***				

National Childhood Cancer Registry (RENCI), 2007–2023.

**Fig. 3:**
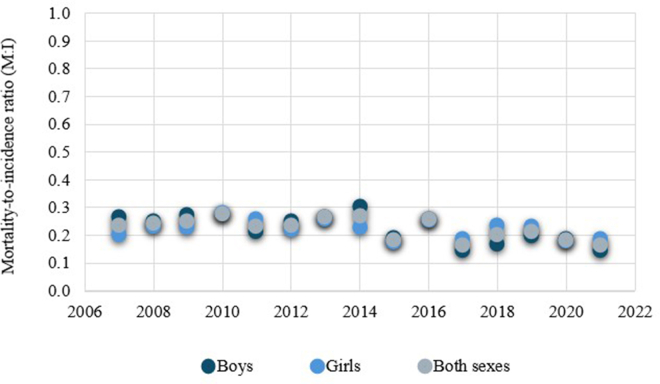
Mortality-to-Incidence (M:I) ratio by calendar year and sex, RENCI, Chile, 2007–2021. Note: M:I ratio as an IARC quality indicator of registry completeness and survival; lower values denote better case ascertainment. Data from RENCI linked to DEIS mortality records.

The Mortality-to-Incidence (M:I) ratio was 0.22 (range 0.16–0.27), a finding compatible with the registry quality indicators and the survival estimates reported in this study (Fig. 3).

Comparability was ensured through the use of standardised international classifications ICCC-3^10^^,^^29^^,^^30^ and ICD-O-3.^9^

The distribution of diagnoses remained stable over time, with leukaemias accounting for 40.4% of cases, central nervous system tumours for 17.6%, and lymphomas for 9.5%, as confirmed by homogeneity tests.

**Fig. 4:**
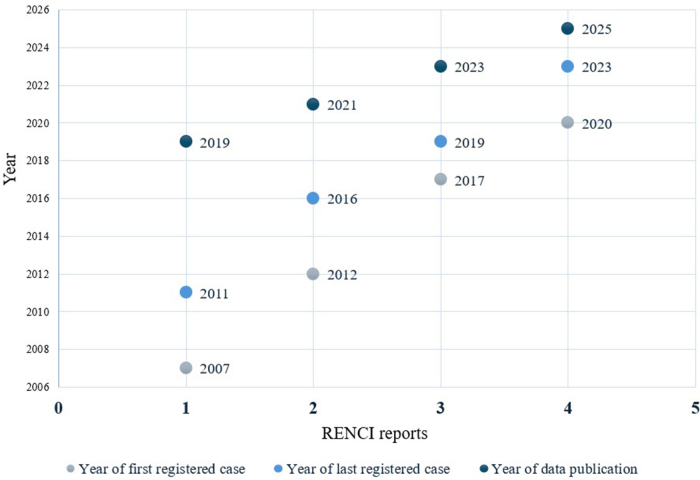
Time gap (in years) between the first and last registered incident case of childhood cancer and the year of publication of each RENCI report: comparison across the four reports. National Childhood Cancer Registry (RENCI), Chile. Note: The time gap represents registry timeliness and was calculated as the interval between the last year of incidence included in each RENCI report and the year of publication. Shorter gaps indicate more timely data collection, validation, linkage, and reporting. Data are based on the four official RENCI reports covering childhood cancer cases diagnosed between 2007 and 2023.

Timeliness improved significantly, with the gap between the data closure period and report publication reduced from 8 years in 2019 to 2 years in 2025^6^^,^^35^^,^^36^ (Fig. 4).

### Policy context for childhood cancer control in Chile

These policies include the National Paediatric Antineoplastic Drugs Programme (PINDA) which has more than 30 years of history and operates through 24 comprehensive and partial care centres nationwide, facilitating access to diagnosis and treatment across the country, unified treatment protocols, and multidisciplinary care teams.^37^

The Explicit Health Guarantees (GES) for children aged 0–14 years with cancer ensures universal coverage and free care from the point of diagnostic suspicion and operates within a framework of nationally defined clinical guarantees and treatment pathways.^38^

In addition, the training of paediatric oncologists and the development of paediatric oncology nursing in Chile,^39^ now recognised as a specialty, form part of the childhood cancer control framework. For decades, training programmes for paediatric oncologists and related subspecialties, particularly nursing, have been supported by clinical practice guidelines (CPGs).^12^^,^^13^^,^^39^, ^40^, ^41^

## Discussion

The RENCI surveillance system provides national epidemiological data on paediatric cancer in Chile for 2007–2023, including incidence, mortality, survival, and the distribution of diagnoses in children aged 0–14 years.

The incidence of paediatric cancer in Chile and its increase over time (AAPC 1.36) are consistent with global trends, with leukaemias, central nervous system (CNS) tumours and lymphomas as the predominant diagnostic groups.^29^^,^^30^

The overall age-standardised incidence rate (ASR) of paediatric cancer in Chile (142.4 per million children aged 0–14 years) falls within the upper range of global estimates reported by IARC for the period 2001–2010 (global average ASR 140.6 per million) and is comparable to figures observed in high-income regions such as North America (157.6 per million) and Europe (143.8–170.8 per million).^29^ It is substantially higher than rates reported in many parts of Asia (87.5–140.9 per million) and Africa (56.3–110.9 per million).^29^

These comparatively higher rates in Chile and other high-income settings may reflect differences in case detection, registration practices, epidemiological surveillance systems, and access to diagnostic resources. Such factors should be considered when comparing incidence estimates across settings with varying levels of registry development and healthcare infrastructure.^30^

Compared with neighbouring Argentina, where childhood cancer incidence averages around 130 per million children aged 0–14 years,^5^ Chile's RENCI data suggest slightly higher overall incidence in Chile (137.4 to 143.0 per million). However, comparisons between countries should be interpreted cautiously, as observed differences may reflect variations in registry practices, case ascertainment, demographic structure, and other contextual factors.^4^^,^^5^

The observed increase in childhood cancer incidence should be interpreted in the context of Chile's advanced demographic transition^42^ and improvements in case detection. The progressive reduction in the paediatric population under 15 years of age, together with potential changes in diagnostic practices, should be considered when evaluating temporal trends. Distinguishing the relative contribution of demographic changes, diagnostic practices, and possible changes in disease occurrence remains beyond the scope of this descriptive study. In particular, leukaemias in Chile (ASR 58.6 per million in RENCI) are within the upper range reported globally, especially in high-income countries (approximately 46–60 per million), while CNS tumours (ASR 24.8) and lymphomas (ASR 12.7) also follow patterns reported by population-based childhood cancer registries in high-income countries.

No clear geographical pattern was observed in incidence by region of residence, as regions exceeding the national rate were located in both northern and southern Chile. This finding suggests that the burden of childhood cancer is distributed across the country rather than concentrated in a specific geographic area. However, regional comparisons should be interpreted with caution because childhood cancer is a relatively rare disease and annual case numbers are small, which may result in random variation in regional rates.

The epidemiological profile of paediatric cancer in Chile during 2007–2023 was broadly similar to that reported by population-based childhood cancer registries in high-income countries: leukaemias accounted for approximately 40% of cases, central nervous system tumours around 18%, and lymphomas for nearly 10%. A clear male predominance was observed, particularly for leukaemias and lymphomas (higher rates in boys), whereas renal and epithelial tumours showed higher incidence in girls.

Children aged 0–4 accounted for more than 42% of all cases, driven mainly by embryonal tumours, whereas the 10–14 years age group showed a transition towards sarcomas and more “adult-type” tumours, including epithelial and germ cell (Supplementary Material S2).

The low proportion of unspecified tumours is consistent with quality indicators recommended by IARC, and the distributions by tumour type, age, and sex are consistent with those reported by other population-based childhood cancer registries.

Five-year survival reached 80.5% (95% CI 76.4–83.9) in the 2020–2023 cohort. For context, the WHO Global Initiative for Childhood Cancer has established a target of at least 60% survival by 2030.^43^ Survival estimates for hepatic tumours and retinoblastoma also improved, although wide confidence intervals due to small numbers limit precision in some subgroups.

Improvements in five-year survival were observed for retinoblastoma (from 82.4% [95% CI 71.5–89.5] in 2007–2011 to 100.0% in 2017–2019 and 2020–2023) and hepatic tumours (from 59.0% [95% CI 42.8–72.0] in 2007–2011 to 86.5% [95% CI 72.5–93.7] in 2020–2023). For hepatic tumours, a stable predominance of hepatoblastoma (approximately 80% of cases in children aged 0–4 years) was observed alongside a slight decline in hepatic carcinomas. These trends were observed during a period in which standardised care pathways and nationwide surveillance systems were in place.^6^^,^^7^^,^^37^^,^^44^

Although mortality showed a downward trend during the study period, this change did not reach statistical significance and should be interpreted with caution. This pattern was observed during a period that also included the implementation of national initiatives such as PINDA and GES^37^^,^^38^^,^^45^; however, the descriptive design of this study does not allow causal inferences regarding the relationship between these initiatives and the observed mortality outcomes.

The observed mortality rates are lower than the global average and consistent with those reported in countries with a high Human Development Index.^45^, ^46^, ^47^

Mortality trends in childhood cancer were broadly similar in Chile and Argentina. Argentina showed a somewhat steeper decline, with an AAPC of approximately −0.9%, reaching around 39.8 per million by 2016.^5^ Despite these favourable trends, both countries continue to face challenges in rural areas, as highlighted in PAHO reports.^48^

The very low proportion of death certificate-only cases (DCO% 0.7%) observed in RENCI is consistent with quality indicators recommended by the International Agency for Research on Cancer (IARC).^11^

Registry quality indicators were consistent with IARC standards,^11^ including 100% population coverage, a microscopically verified rate (MV%) of 93.5%, and a death certificate-only rate (DCO%) of 0.7%.

Timeliness also improved markedly, bringing reporting timelines closer to those reported by established registries in high-income countries.^49^

The data generated by RENCI may support future observational and health services research, including studies of environmental and genetic factors associated with childhood cancer, linkage with long-term health records to assess treatment outcomes and survivorship, and evaluations of established models of care.^37^^,^^38^^,^^50^ Such analyses may provide additional evidence relevant to childhood cancer surveillance and to monitoring progress towards the objectives of the WHO Global Initiative for Childhood Cancer (CureAll).^43^

### Limitations

This study has several limitations. First, because of its descriptive observational design, associations between the policy context described and the observed trends in incidence, survival, and mortality are ecological and temporal in nature and should not be interpreted as evidence of causal effects of specific policies or interventions.

Although the National Childhood Cancer Registry (RENCI) achieves 100% population coverage, several limitations inherent to routinely collected registry data remain. Physicians contribute to the registry largely during unpaid hours and without protected time specifically allocated to this task, which poses a risk to the long-term sustainability of the system and may increase the likelihood of future under-registration.

A further limitation is that, despite the high microscopic verification rate (MV% 93.5%), some degree of under-registration may still occur in certain tumour types, particularly central nervous system neoplasms, including low-grade and benign/in situ tumours that are often diagnosed by imaging alone without histological confirmation, and retinoblastoma, both of which are internationally recognised as being at greater risk of under-ascertainment.^29^ To mitigate this risk, information is cross-referenced with multiple complementary sources, including the Death Registry (DEIS), hospital discharge databases, SIGGES, Hospital and Population-Based Cancer Registries, civil registry queries and historical RENCI queries, as detailed in the flow diagram of the validation process (Supplementary Material S1).

Another limitation inherent to population-based cancer registries is the potential instability of annual incidence rates due to the rarity of paediatric cancer and the resulting small number of cases per year. This may generate year-to-year fluctuations; accordingly, the use of aggregated 5-year periods is recommended for more robust and internationally comparable analyses.

Some sex-stratified estimates, particularly for rare diagnostic groups, should be interpreted cautiously because of the small number of events.

The decline in incidence observed in 2023 relative to 2021 and 2022 is noteworthy and may reflect underdiagnosis and, to a lesser extent, under-registration. Although the risk of under-registration is minimised through systematic cross-validation with multiple data sources, continued monitoring of future trends will be essential.

In addition, the incomplete and relatively short follow-up for the most recent cohorts (2020–2023) represents an important limitation and may lead to underestimation or overestimation of survival in certain tumour types. Survival estimates for the 2020–2023 cohort should be interpreted cautiously because complete five-year follow-up is not yet available.

The mortality-to-incidence (M:I) ratio was used as an IARC quality indicator and as a descriptive measure of the relationship between mortality and incidence. However, in paediatric oncology, this metric may be influenced by deaths occurring among cases diagnosed in previous years, including late relapses and long-term treatment-related complications, which limits its ability to reflect the survival experience of recently incident cases alone. The low M:I ratio observed during the study period was consistent with the favourable survival estimates reported by RENCI. Although the M:I ratio has been widely used as a descriptive indicator in population-based cancer studies, it should be interpreted with caution, as it provides only an indirect measure of the relationship between incidence and mortality and does not constitute direct evidence of the effects of specific health policies or interventions.

Although challenges remain in securing resources for continuous training of registering physicians and technological infrastructure updates, which could affect the timeliness and completeness of the registry in the long term, data validation, record linkage, and quality control procedures remain integral components of registry operations.

Finally, as with most population-based registries, RENCI lacks detailed clinical variables such as stage at diagnosis, molecular risk stratification, cytogenetic characteristics, and specific treatment protocols. The inclusion of such information would greatly enhance the depth of future analysis of outcomes.

### Conclusion

Chile's National Childhood Cancer Registry (RENCI) represents one of the few nationwide population-based childhood cancer registries in Latin America and provides long-term epidemiological surveillance for the period 2007–2023. This study reports increasing incidence, improved five-year survival in recent cohorts reaching 80.5%, and a non-significant downward trend in mortality. National childhood cancer surveillance is supported by an established registration infrastructure, including the DHIS2 platform.^51^^,^^52^ The experience of RENCI may be useful for other low- and middle-income countries seeking to strengthen childhood cancer registration, surveillance, and control systems, in line with the objectives of the WHO Global Initiative for Childhood Cancer (CureAll).

## Contributors

PC and KN conceived the study, designed the methodology, and verified the underlying data. PC conducted the statistical analyses and drafted the manuscript. KN contributed to survival analyses and data validation. MP and MS contributed to data consolidation, validation, and bibliographic review. JP and JV contributed to interpretation of the findings and critically revised the manuscript. All authors had full access to the study data, approved the final manuscript, and accepted responsibility for the decision to submit for publication.

## Data sharing statement

Individual-level data used in this study are not publicly available due to legal and confidentiality restrictions governing health information. Requests for access to aggregated data should be directed to the corresponding author and will be considered in accordance with Chilean Ministry of Health regulations and applicable data protection requirements.

## Declaration of interests

None declared.
