# Supplementary material for: The National Childhood Cancer Registry of Chile: a population-based study of childhood cancer and insights for low- and middle-income countries
Source: Lancet Reg Health Am. 2026 Aug 7;62:101588. doi: 10.1016/j.lana.2026.101588 (PMC13476546; doi:10.1016/j.lana.2026.101588)
Supplement: Supplementary file 1 [file mmc1.pdf]

Supplementary Materials

The National Childhood Cancer Registry of Chile: a population-based study of childhood cancer and insights for low- and middle-income countries

Supplementary Figure S1. Validation process of the National Childhood Cancer Registry (RENCI), Chile. 2

Supplementary Table S2. Incidence by ICCC-3 group, sex and age subgroup 3

Supplementary Figure S1. Validation process of the National Childhood Cancer Registry (RENCI), Chile.

Flow diagram of case ascertainment, validation, record linkage, data enrichment, and quality control in the National Childhood Cancer Registry (RENCI), Chile, 2007–2023.

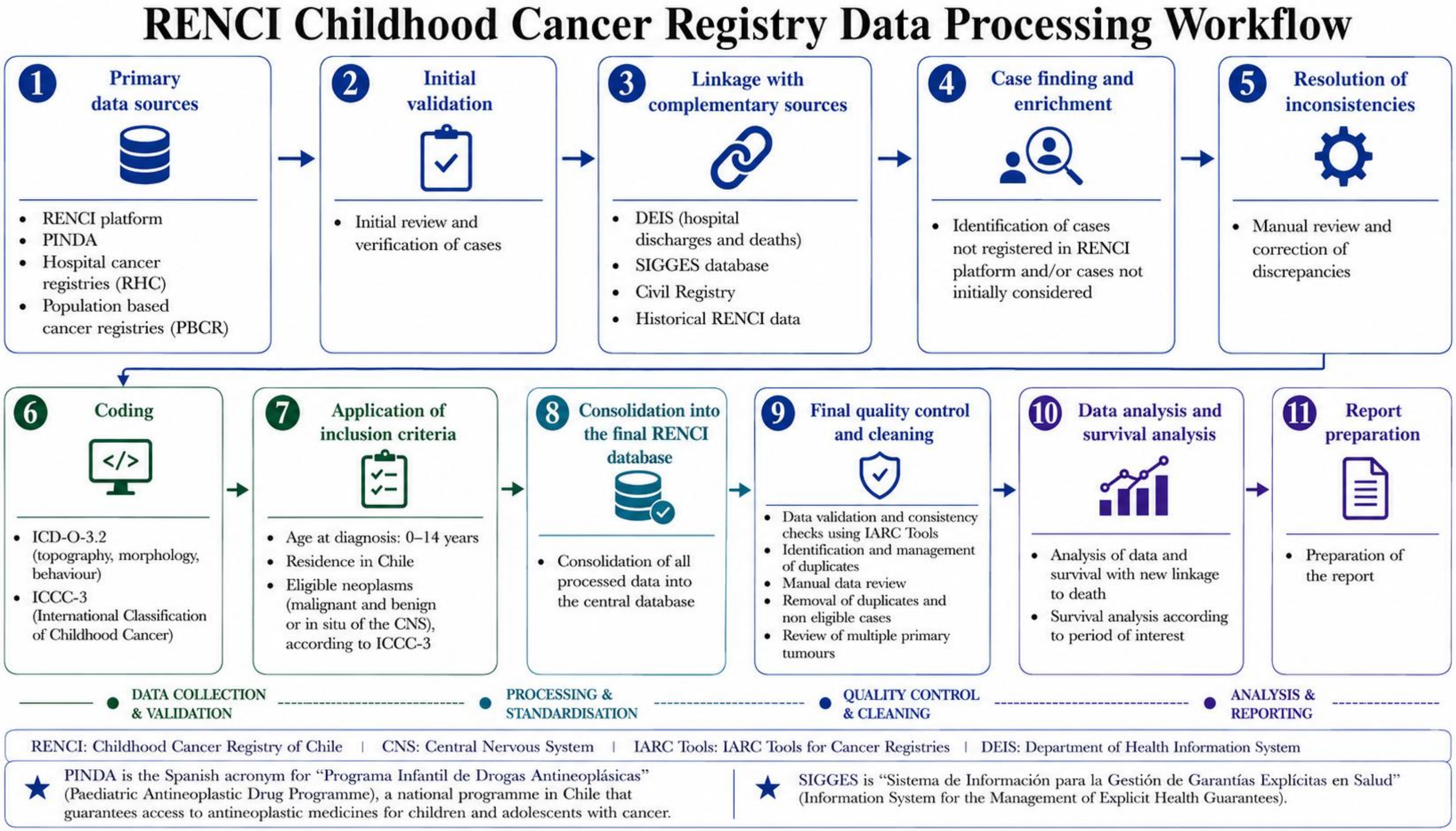

Flow diagram depicting the sequential steps from primary data sources (RENCI platform, PINDA, hospital cancer registries [RHC], and population-based cancer registries [RPC]) through initial validation, linkage with complementary sources (DEIS, hospital discharges, SIGGES, civil registry queries, historical RENCI queries), case finding and enrichment, resolution of inconsistencies, coding with ICD-O-3.2 and ICCC-3, application of inclusion criteria (age 0–14 years at diagnosis, residence in Chile, eligible neoplasms), consolidation into the final RENCI database, and final quality control and cleaning using IARC-Tools and data review. Duplicates, multiple primary tumours, and non-eligible cases were removed at appropriate steps. **AI statement:** ChatGPT (OpenAI) was used to generate this figure based on author-provided instructions describing the workflow of the National Childhood Cancer Registry of Chile (RENCI), including data collection, record linkage, validation procedures, and quality assurance processes**.** The authors reviewed, edited, and approved the output and take full responsibility for its content.

Supplementary Table S2. Incidence by ICCC-3 group, sex and age subgroup

Number of incident cases and incidence rates of childhood cancer by ICCC-3 diagnostic group, sex, and age subgroup. National Childhood Cancer Registry (RENCI), Chile, 2007–2023.

ICCC-3 Group	Cases (n)			Total (n)	Age-specific incidence rate			Total crude incidence rate	Total age-standardised incidence rate
	**0-4 years**	**5-9 years**	**10-14 years**	**0-14 years**	**0-4 years**	**5-9 years**	**10-14 years**	**0-14 years**	**0-14 years**
**Boys**									
I. Leukaemias, myeloproliferative diseases, and myelodysplastic diseases	933	570	476	1 979	88·7	52·9	42·9	61·1	63·9
II. Lymphomas and reticuloendothelial neoplasms	118	215	233	566	11·2	20·0	21·0	17·5	16·9
III. CNS and miscellaneous intracranial and intraspinal neoplasms	310	280	235	825	29·5	26·0	21·2	25·5	26·0
IV. Neuroblastoma and other peripheral nervous cell tumours	146	25	3	174	13·9	2·3	0·3	5·4	6·2
V. Retinoblastoma	114	10	0	124	10·8	0·9	0·0	3·8	4·5
VI. Renal tumours	108	23	7	138	10·3	2·1	0·6	4·3	4·8
VII. Hepatic tumours	78	19	11	108	7·4	1·8	1·0	3·3	3·7
VIII. Malignant bone tumours	23	70	155	248	2·2	6·5	14·0	7·7	7·0
IX. Soft tissue and other extraosseous sarcomas	121	78	93	292	11·5	7·2	8·4	9·0	9·2
X. Germ cell tumours, trophoblastic tumours, and neoplasms of gonads	97	29	130	256	9·2	2·7	11·7	7·9	7·8
XI. Other malignant epithelial neoplasms and malignant melanomas	4	21	79	104	0·4	1·9	7·1	3·2	2·8
XII. Other and unspecified malignant neoplasms	13	0	6	19	1·2	0·0	0·5	0·6	0·6
Total	2 065	1 340	1 428	4 833	196·4	124·4	128·8	149·3	153·6
**Girls**	**0-4 years**	**5-9 years**	**10-14 years**	**0-14 years**	**0-4 years**	**5-9 years**	**10-14 years**	**0-14 years**	**0-14 years**
I. Leukaemias, myeloproliferative diseases, and myelodysplastic diseases	741	466	376	1 583	73·2	44·9	35·1	50·7	53·0
II. Lymphomas and reticuloendothelial neoplasms	60	85	125	270	5·9	8·2	11·7	8·7	8·3
III. CNS and miscellaneous intracranial and intraspinal neoplasms	265	259	201	725	26·2	24·9	18·8	23·2	23·6
IV. Neuroblastoma and other peripheral nervous cell tumours	115	23	5	143	11·4	2·2	0·5	4·6	5·2
V. Retinoblastoma	117	4	2	123	11·6	0·4	0·2	3·9	4·7
VI. Renal tumours	141	32	9	182	13·9	3·1	0·8	5·8	6·6
VII. Hepatic tumours	57	4	8	69	5·6	0·4	0·7	2·2	2·5
VIII. Malignant bone tumours	25	60	153	238	2·5	5·8	14·3	7·6	7·0
IX. Soft tissue and other extraosseous sarcomas	92	48	86	226	9·1	4·6	8·0	7·2	7·3
X. Germ cell tumours, trophoblastic tumours, and neoplasms of gonads	36	44	87	167	3·6	4·2	8·1	5·4	5·1
XI. Other malignant epithelial neoplasms and malignant melanomas	11	33	197	241	1·1	3·2	18·4	7·7	6·8
XII. Other and unspecified malignant neoplasms	6	0	15	21	0·6	0·0	1·4	0·7	0·6
**Total**	1 666	1 058	1 264	3 988	164·6	101·9	118·2	127·8	130·9
**Both sexes**	**0-4 years**	**5-9 years**	**10-14 years**	**0-14 years**	**0-4 years**	**5-9 years**	**10-14 years**	**0-14 years**	**0-14 years**
I. Leukaemias, myeloproliferative diseases, and myelodysplastic diseases	1 674	1 036	852	3 562	81·1	49·0	39·1	56·0	58·6
II. Lymphomas and reticuloendothelial neoplasms	178	300	358	836	8·6	14·2	16·4	13·1	12·7
III. CNS and miscellaneous intracranial and intraspinal neoplasms	575	539	436	1 550	27·9	25·5	20·0	24·4	24·8
IV. Neuroblastoma and other peripheral nervous cell tumours	261	48	8	317	12·6	2·3	0·4	5·0	5·7
V. Retinoblastoma	231	14	2	247	11·2	0·7	0·1	3·9	4·6
VI. Renal tumours	249	55	16	320	12·1	2·6	0·7	5·0	5·7
VII. Hepatic tumours	135	23	19	177	6·5	1·1	0·9	2·8	3·1
VIII. Malignant bone tumours	48	130	308	486	2·3	6·1	14·1	7·6	7·0
IX. Soft tissue and other extraosseous sarcomas	213	126	179	518	10·3	6·0	8·2	8·1	8·3
X. Germ cell tumours, trophoblastic tumours, and neoplasms of gonads	133	73	217	423	6·4	3·5	10·0	6·7	6·5
XI. Other malignant epithelial neoplasms and malignant melanomas	15	54	276	345	0·7	2·6	12·7	5·4	4·8
XII. Other and unspecified malignant neoplasms	19	0	21	40	0·9	0·0	1·0	0·6	0·6
**Total**	3 731	2 398	2 692	8 821	180·8	113·4	123·6	138·8	142·4

Note: Number of incident cases, crude incidence rates, and age-standardised incidence rates (Segi world standard population) of childhood cancer by ICCC-3 group, sex, and age group. National Childhood Cancer Registry (RENCI), Chile, 2007–2023. Rates per million children per year.
